# Moral injury and its effects on academic performance of student nurses in South Africa

**DOI:** 10.4102/curationis.v48i1.2697

**Published:** 2025-04-30

**Authors:** Kwanele Mbazo, Richard M. Rasesemola, Gugu Ndawo

**Affiliations:** 1 Department of Nursing, Faculty of Health Sciences, University of Johannesburg, Johannesburg, South Africa

**Keywords:** moral injury, moral beliefs, student nurse, academic performance, healthcare professionals

## Abstract

**Background:**

Morals are norms of conduct, behaviour and guidelines that nurses must uphold and observe in nursing. Moral codes require nurses and student nurses to respect human rights and dignity, and act with sound ethical judgement. However, when student nurses witness and fail to prevent acts that transgress their deeply held moral beliefs, their moral code is damaged, and deep emotional wounds ensue, leading to moral injury.

**Objectives:**

This study aimed to investigate the prevalence and effects of moral injury in student nurses at a higher education institution in South Africa.

**Method:**

A quantitative, non-experimental cross-sectional survey was relied upon. The total population sampling method was applied, resulting in 124 respondents. Data were collected among the undergraduate student nurses registered at a higher education institution in Johannesburg from first to final year using a self-administered Moral Injury Symptom Scale – Healthcare Professionals questionnaire. Data were analysed using IBM Statistical Package for the Social Sciences (SPSS) Statistics version 28 software.

**Results:**

The results indicated that more than a third (34%) of students experienced moral injury. Furthermore, a positive significant relationship between the presence of moral injury and academic performance among the students was noted (*p* = 0.029).

**Conclusion:**

In this study, the prevalence of moral injury among student nurses was investigated and its effects on academic performance were reported.

**Contribution:**

The effects of moral injury among student nurses indicate a need for higher education institutions to design and implement nursing curriculum that would assist students to develop moral resilience and ethical behaviour.

## Introduction

Florence Nightingale was distinct about the need for morals and emphasis on ethical theory in nursing. This was reflected in the selection criteria used to recruit ideal nurses. These nurses had to embody truthfulness, loyalty, honesty, devotion and humility. Overall, they were required to be noble and morally aware with the ability to serve society with respect (Glasper 2020; Thompson & Darbyshire 2020). Morals are built and extended from foundations such as religion, cultural frames, political affiliation and social conservatism (Mooijman, Meindl & Graham 2020; Silver & Silver 2021). Van der Cingel and Brouwer (2021) define morals as norms of conduct, behaviour and guidelines that nurses must uphold and observe in nursing. Student nurses are not exempted because education and training to maintain and observe such norms begin when they enter the field and continue throughout (Poorchangizi et al. 2019; Yoder et al. 2022).

Moral injury was conceptualised initially by Jonathan Shay in 1994. Moral injury can be defined as the psychological, emotional and existential harm that occurs when an individual experiences a cognitive conflict between their internal moral belief system and the actions witnessed or engaged in (Dickinson 2023; Mewborn et al. 2023; Sugrue 2020; Wang et al. 2020). A person with moral injury presents with symptoms of guilt, anxiety, shame, anger and depression. These may lead to profound demoralisation, loss of trust and existential fear. These symptoms are long-lasting and may disrupt a person’s confidence, expectations about their own or others’ motivation and the ability to behave in an ethical and just manner (Dickinson 2023; Mewborn et al. 2023; Sugrue 2020).

Visser (2020) recognised the need to address moral injury among healthcare professionals and within the South African context. Moral injury has been researched vastly among military personnel and has gradually increased awareness among healthcare professionals because of the threats to their mental health. Murray, Krahé, and Goodsman (2018) identified moral injury among medical students and stated the necessity for further exploration to understand the symptoms of individuals that are subsyndromal for post-traumatic stress disorder.

As mentioned, student nurses come into nursing with morals that are often interpreted as avoiding the infliction of harm and doing wrong to the ill and those in need. Therefore, they view themselves as helpers; kind and understanding individuals (Van der Cingel & Brouwer 2021). Professional guidance and ongoing conversations on ethics become imperative for them in the clinical setting. Such imperativeness compels their supervisors to portray exemplary behaviour that seeks to avoid the infliction of harm or wrongdoing on the ill (Zolkefli 2021).

Student nurses are more likely to sustain moral injury because of their limited clinical experience, capacity for clinical judgement and vulnerability, which present obstacles to engaging in the challenges of the clinical environment. Due to their limited clinical experience, student nurses are frequently unable to comprehend the clinical situation, further increasing feelings of distress and inadequacy (Bektas et al. 2021; Martin, Kam & Aziz 2021; Murray, Sundin & Cope 2019). Hossain and Clatty (2021) state that student nurses face high stakes, decision-making challenges that affect both their clinical environment experience and personal lives. Therefore, this study aimed to investigate the prevalence and effects of moral injury among student nurses at a higher education institution (HEI) in Gauteng, South Africa.

### Problem statement

Student nurses witness cases of adverse treatment directed towards patients in the clinical facilities where they are placed for experiential learning. Examples of such cases include a mother who gave birth on the floor in one of government hospitals in South Africa without the assistance of healthcare professionals (Phetho 2021). Another one is of a patient who was not fed for more than 100 h and was allegedly accommodated in the same room with corpses that had not yet been transferred to the mortuary. This patient later died, but days before his death, he uploaded his experience of healthcare professionals’ negligence and ill-treatment in a series of posts on a social media platform (Mathe 2021). In most instances, there is always little or nothing that the student nurses can do during such incidents. When student nurses witness or fail to prevent these acts of negligence that transgress their deeply held moral beliefs, their moral code is damaged, leaving deep emotional wounds, which may be characterised by feelings of guilt, shame, anger, self-condemnation, existential crisis and alienation or social withdrawal (Hebert 2020). They may develop feelings that may alter their self-concept for life (Cartolovni et al. 2021). They may end up not comprehending what is happening or how to deal with the resulting emotions. Therefore, failure to prevent these incidences or protect student nurses from witnessing them may lead to the emergence of moral injury and related near misses and medical errors as they transition into clinical practice as professional nurses (Murray et al. 2019). While they may not be able to render sufficient nursing care because of constraints beyond their control, student nurses may also be faced with the challenge of acquiring the academic knowledge related to the patients’ needs because of moral injury. Thus, in essence, these challenges in the healthcare system place a burden on student nurses and contribute to the virtual cycle of poor patient care and health outcomes in South Africa.

### Research aim

This study aimed to investigate the prevalence and effects of moral injury in student nurses of a HEI in Gauteng, South Africa.

## Research design and methods

### Research design

Quantitative, non-experimental, descriptive, and cross-sectional research designs were used in this study. In this study, a quantitative research approach was employed to systematically investigate the prevalence and effect of moral injury among nursing students at a HEI. This approach, which is formal, objective and systematic, allowed for the generation of numerical data about the world, thereby enhancing the objectivity of the research (Gray & Grove 2021; Indu & Vidhukumar 2020). A non-experimental research design enabled the researcher to collect data without introducing treatments or making changes to the participants’ surroundings, thereby maintaining the natural environment of the nursing students (Gray & Grove 2021; Polit & Beck 2020). The study also employed a descriptive research design, which used numbers to provide an accurate account of the characteristics of the phenomena, thereby providing numerical evidence of the occurrence of moral injury (Gray & Grove 2021; Polit & Beck 2020). A cross-sectional survey was conducted among 162 nursing students at an HEI in Gauteng. This survey examined data at one point in time, allowing the researcher to collect data in one setting and at the same time using all the nursing students in different year groups. The participants were administered a once-off self-report questionnaire, without any prior exposure or follow-up, enabling the researcher to capture a snapshot of the phenomenon of moral injury within a single data collection period (Gray & Grove 2021; LoBiondo-Wood & Haber 2021).

### Research setting

This study took place at a HEI (University) in Gauteng, South Africa. The participants were enrolled in an undergraduate 4-year degree in nursing which would lead them to qualify as professional nurses and midwives, and immediately qualify graduates to practise as professional nurses in the government healthcare facilities.

### Population and sampling

The study population consisted of undergraduate student nurses who were undergoing education and training at a HEI in Gauteng, South Africa. All 162 student nurses across four levels of study registered at this HEI were accessible to the researcher.

A total population sampling method, which is a purposive sampling technique focussing on the whole population of interest, was relied upon in this study (De Jesus & Buenaventura 2021). This sampling method was preferred as the population size remains relatively small.

### Data collection

Data were collected using a modified and adapted version of *the Moral Injury Symptom Scale – Healthcare Professionals (MISS-HP)* questionnaire developed by Mantri et al. (2020). The data collection tool was divided into six sections (A–F). Section A gathered demographic data, while sections B-F assessed moral injury, moral distress, depression, anxiety and academic performance. The questionnaire, comprising 54 items and utilising a 5-point Likert scale (1-Strongly disagree, 2-Agree, 3-Neither disagree, 4-Agree, & 5-Strongly agree), was adapted to incorporate five constructs that measured moral injury, moral distress, depression, anxiety and academic performance. Section B contained 10 items pertaining to moral injury, adapted from Koenig et al. (2020). Sections C–F, which required no adaptation permission, included 15 statements measuring moral distress (adapted from Corley et al. 2001) and eight items assessing depression symptoms (adapted from the PHQ-9 by Koenig et al. [2020]), excluding the question on suicidal ideation.

The adopted and modified Moral Injury Symptom Scale – Healthcare Professionals version (MISS-HP) scale was critiqued following the approaches of face validity, content validity and construct validity. The researcher employed Exploratory Factor Analysis (EFA) to ascertain whether the questionnaire items measured the intended constructs and to examine the interrelationships among the items. Reliability analysis was conducted to assess the consistency of the measurement scales, with high reliability indicating consistent results. The Cronbach alpha coefficients for the constructs were: moral injury (α = 0.63), moral distress (α = 0.79), depression (α = 0.84), anxiety (α = 0.89) and poor academic performance (α = 0.77). The α values for depression and anxiety were deemed strong, while those for moral distress and poor academic performance were moderate. The α for moral injury was considered low yet acceptable. Gagnon (2019) suggests continuous scale refinement to improve a scale, and low-reliability coefficients (< 0.60) indicate weak instrument dependability. Overall, the adapted moral injury scale demonstrated dependability and consistency, suggesting its applicability to other participant samples to yield comparable results.

After relevant ethical clearance and permissions to include nursing students in the study were obtained from the University of Johannesburg, Faculty of Health Sciences Research Ethics Committee (REC-1332-2021) and the Division for Institutional Planning, Evaluation and Monitoring (DIPEM), the researcher approached all 162 potential participants. The researcher distributed 155 questionnaires among potential participants who provided consent; 124 questionnaires were completed and returned to the researcher, resulting in a response rate of 80%.

The ethical principle of justice was upheld, where all respondents had an equal chance of being selected. Informed consent included oral and written information, and final consent was provided in writing. Other ethical principles such as autonomy, beneficence and non-maleficence were also all adhered to. The researcher provided information and contacts of the Centre for Psychological Services and Career Development (PsyCaD) for debriefing, counselling and psychological support for the respondents in cases where they could have experienced psychological or emotional discomfort.

### Data analysis

Data analysis was done using IBM Statistical Package for the Social Sciences (SPSS) software version 28.0 to perform descriptive and inferential statistics with the help of a qualified statistician. The statistical tests included EFA, the Kaiser-Meyer-Olkin test, Bartlett’s Test of Sphericity, and Analysis of Variance (ANOVA) tests.

### Ethical considerations

Ethical clearance and permissions to include nursing students in the study were obtained from the University of Johannesburg, Faculty of Health Sciences Research Ethics Committee (REC-1332-2021) and the DIPEM. Informed consents were obtained from each student before they participated in the study.

## Results

Results include demographic data of the respondents, the prevalence of moral injury and the prevalence of poor academic performance.

### Demographic information

Demographic information presented in Table 1 includes the respondents’ age group, gender and level of study.

**TABLE 1:** Demographic information.

Demographic factor	Categories	Frequency	%
Age group (years)	18–21	70	55.6
	22–25	46	37.0
	26–29	7	5.6
	30–34	1	0.8
Gender	Male	24	19.0
	Female	100	81.0
Level of study	First	37	29.8
	Second	26	21.0
	Third	30	24.2
	Final	31	25.0

The mean age of the respondents who completed the survey was 21.4 years. More than half (55.6%, *n* = 70) of the respondents fell under the age category of 18–21 years. At the same time, the age category of 30 years to 34 years was represented by the least number of respondents at 0.8% (*n* = 1). Regarding gender distribution, 81.0% (*n* = 100) of the respondents were female. The distribution of the sample according to their level of study was as follows: respondents from first year were more than any other study level respondents at 29.8% (*n* = 37). All other year’s groups were almost evenly distributed.

### Prevalence of moral injury

The prevalence of moral injury is depicted with the frequency count, percentage responses, mean and standard deviations for each item (Table 2).

**TABLE 2:** Prevalence of moral injury.

Statement related to moral injury			Disagree strongly/disagree	Neither disagree nor agree	Agree/ strongly agree	Mean	s.d.
1	I feel betrayed by other health professionals whom I once trusted.	*n*	57	28	39	2.67	1.194
		%	46.0	22.6	31.5		
2	I feel guilty over failing to save someone from being seriously injured or dying.	*n*	32	13	79	3.51	1.346
		%	25.8	10.5	63.7		
3	I feel ashamed about what I’ve done or not done when providing care to my patients.	*n*	32	23	69	3.40	1.160
		%	25.8	18.5	55.6		
4	I am troubled by having acted in ways that violated my own morals or values.	*n*	38	17	69	3.30	1.288
		%	30.6	13.7	55.6		
5	Most people with whom I work as a health professional are trustworthy.	*n*	25	35	64	3.42	1.052
		%	20.2	28.2	51.6		
6	I have a good sense of what makes my life meaningful as a health professional.	*n*	9	18	97	4.06	0.965
		%	7.3	14.5	78.2		
7	I have forgiven myself for what’s happened to others whom I have cared for.	*n*	23	26	75	3.57	1.061
		%	18.5	21.0	60.5		
8	All in all, I am inclined to feel that I’m a failure in my work as a student nurse.	*n*	81	27	16	2.19	1.047
		%	65.3	21.8	12.9		
9	I sometimes feel God is punishing me for what I’ve done or not done while caring for patients.	*n*	88	17	19	2.05	1.161
		%	71.0	13.7	15.3		
10	Compared to before I went through these experiences, my religious or spiritual faith has strengthened.	*n*	30	34	60	3.36	1.225
		%	24.2	27.4	48.4		

s.d., standard deviation.

Table 2 presents the results of items under the construct of moral injury. Almost a third (*n* = 39) of the respondents agreed with feeling betrayed by other health professionals they once trusted. More than 63.0% (*n* = 69) of respondents reported feelings of guilt over failing to save someone from being seriously injured or dying, and 55.6% (*n* = 69) reported feeling ashamed about what they have done or not done when providing care to their patients. Furthermore, 55.6% (*n* = 69) reported being troubled by having acted in ways that violated their morals and values.

Statements 1 to 4 in Table 2 had an overall positive response concerning the symptoms of moral injury; thus, most of the students agreed to have experienced feelings of guilt, shame, betrayal and moral concerns evidenced by a mean score of above 3 indicating the majority of neutral responses with a standard deviation (s.d.) of 1.1.

Statements 5, 6, 7 and 10 in Table 2 focussed on the loss of trust, meaning, inability to forgive and loss of faith. These items were positively worded, which signified the presence of moral injury when the respondents disagreed with the statements. The results showed that a minority of the participants had negative perceptions of their work and themselves as student nurses. Only 20.3% (*n* = 25) reported that most people with whom they worked as health professionals were not trustworthy. Furthermore, 18.5% (*n* = 23) of the respondents had not forgiven themselves for what happened to the people they cared for, and 24.2% (*n* = 30) disagreed that compared to before they went through these experiences, their religious and spiritual faith had strengthened.

Statements 8 and 9 had the most substantial loadings on self-condemnation and punishment by God. The results further indicate that the later variables were negatively worded; thus, respondents who endorsed these items had a high moral injury. The results suggest that 12.9% (*n* = 16) of the respondents agreed with being inclined to feel that they are failures in their work as health professionals, and 15.3% (*n* = 19) of the respondents agreed that sometimes they feel like God is punishing them for what they have done or not done while caring for patients. These statements had mean (M) values below 3 and a s.d. of 1 (Statement 8: *M* = 2.19, s.d. = 1.047, and Statement 9: *M* = 2.05, s.d. = 1.161), depicting that the majority of the respondents disagreed with the items. The results of this study indicated that moral injury was experienced by more than one-third (34.6%; *n* = 43) of the respondents.

### Academic performance

The results related to participants’ academic performance are presented in Table 3. The results include frequency count, percentage responses, and mean and s.d. for each statement pertaining to academic performance.

**TABLE 3:** Academic performance.

Statements related to academic performance		Strongly disagree/disagree	Neither disagree nor agree	Strongly agree/agree	Mean	s.d.
The course was not what I had expected.	*n*	19	10	95	3.91	1.090
	%	15.3	8.1	76.6		
I find the course too difficult.	*n*	43	38	43	3.07	1.120
	%	34.7	30.6	34.7		
I don’t find the course material interesting.	*n*	79	19	26	2.52	1.100
	%	63.7	15.3	21.0		
The pace and workload are too great.	*n*	39	15	70	3.44	1.264
	%	31.5	12.1	56.5		
I have increased pressure at home and work.	*n*	29	18	77	3.56	1.185
	%	23.4	14.5	62.1		
I experience temporary crises at home and work.	*n*	56	18	50	2.94	1.271
	%	45.2	14.5	40.3		
I always feel isolated studying on my own.	*n*	65	17	42	2.75	1.279
	%	52.4	13.7	33.9		
I underestimated the study time required.	*n*	37	21	66	3.35	1.289
	%	29.8	16.9	53.2		
I do not have the study skills or background knowledge required.	*n*	72	25	27	2.60	1.235
	%	58.1	20.2	21.8	3.13	1.204

s.d.,standard deviation.

The construct of poor academic performance was assessed by nine statements with a high mean score above 3 and a s.d. of 1.204, indicating that the respondents agreed that they faced academic challenges. The results showed that 44.0% (*n* = 55) of the respondents reported to have experienced poor academic performance. The majority (76.6%; *n* = 95) of the respondents reported that the course did not meet their expectations, whereas 34.7% (*n* = 43) of the respondents reported that the course was too challenging. While 21.0% (*n* = 26) of the respondents indicated that they did not find the course material interesting,, more than half (56.5%; *n* = 70) reported that the pace and workload were too great. The questionnaire required the respondents to identify any external factors that might adversely affect their academic achievement. These items showed that more respondents agreed to have increased pressures at home and work, at 62.1% (*n* = 77), and 40.3% (*n* = 50) of the respondents reported experiencing a temporary crisis at home and in clinical practice. Participants who reported feeling isolated and studying on their own comprised of 33.9% (*n* = 42) of the sample, whereas 53.2% (*n* = 66) reported that they underestimated the study time required. Also, a further 21.8% (*n* = 27) reported lacking the study skills or background knowledge needed for the course.

### Correlation between moral injury and academic performance

Pearson’s Product Moment Correlation Coefficient (*r*) was used to measure the direction and the strength of the relationship between moral injury as an independent variable and the dependent variables, including moral distress, depression and poor academic performance. Table 4 illustrates the correlation between moral injury and moral distress, depression, anxiety and poor academic performance.

**TABLE 4:** Correlation analysis of moral injury and academic performance (*N* = 124).

		Moral injury
Academic performance	Pearson correlation	0.196^*^
	Sig. (2-tailed)	0.029

* , Correlation is significant at the 0.05 level (2-tailed).

Academic performance had a positive but weak linear relationship with moral injury, with *r* = 0.196. The *p*-value of 0.029 suggested that there is a statistical significance between the two variables. The presence of moral injury is associated with poor academic performance in student nurses at an HEI.

## Discussion

The results revealed that there is moral injury among student nurses; 34.6% of the respondents had moral injury. In this study, moral injury was investigated using a questionnaire that contained statements that had a more substantial loading for guilt, shame, betrayal and moral concerns, which depicted moral injury symptoms. Almost a third of the participants in this study reported feeling betrayed by other health professionals whom they once trusted. Other statements included in the questionnaire to investigate moral injury focussed on the respondents’ loss of trust, loss of meaning, inability to forgive and loss of faith. In this study, almost a fifth of the respondents reported moral injury symptoms pertaining to the religiosity aspect of moral injury.

Moral injury is prevalent among healthcare professionals, and it is more prevalent in some regions than others (Rushton et al. 2022; Zerach & Levi-Belz 2021). In China, moral injury affected 20.0% of healthcare professionals (Zhizhong et al. 2020). While in American healthcare workers, moral injury affected 7.8% of the healthcare workers (Mantri et al. 2020). Nurses are at a higher risk of developing moral injury when compared with other healthcare professional members because of long hours and the ethical dilemmas they encounter in their work as well as the physical, emotional and psychological challenges when dealing with patients. (Rushton et al. 2022). Zerach and Levi-Belz (2021) also confirmed that more than 43.0% of nurses in their study had moral injury. Student nurses, who form part of the healthcare professional teams (Leburu 2020), by virtue of their novice status and the lack of mentorship, are at a higher risk of developing moral injury (Murray et al. 2018). Student nurses tend to experience high levels of self-criticism and low levels of self-compassion, which are common traits among individuals who suffer from moral injury (Zerach & Levi-Belz 2021). As members of the healthcare team, student nurses collaborate with senior-level professional nurses who provide constant guidance, feedback and continuous supervision (Quade, Bonner & Greenbaum 2020). However, because of limited expertise in coping with potentially moral injurious events in the clinical setting, they are at a higher risk of developing moral injury (Murray et al. 2018).

Younger age is another risk factor related to the propensity to develop moral injury. Novice nurses are more vulnerable to violations during clinical practice, increasing the probability of experiencing moral injury (Spilg et al. 2022; Zhizhong et al. 2020). Violations refer to incidents and challenges within the healthcare system that students may encounter in the clinical environment. Perhaps immature coping strategies or occupational-related psychological distress that arise from the ethical dilemmas faced by nurses in a clinical environment mediate the results (Hassanzadeh Naeini et al. 2020; Mantri et al. 2020). That is, limited clinical experience and trained capacity for clinical judgement among student nurses present obstacles in managing the challenges of the clinical environment (Bektas et al. 2021; Martin et al. 2021; Murray et al. 2019). Student nurses may not be able to comprehend what is happening when they witness and fail to respond to a situation where a supervisor engages in amoral and dishonourable behaviour (Quade et al. 2020). Student nurses may end up adopting unethical or unprofessional behaviour themselves (Koenig et al. 2020; Mewborn et al. 2023) or they may end up with feelings of distress and inadequacy.

In addition to being a nurse and being of younger age, other authors identified gender as a risk factor for moral injury. Female nursing professionals were more likely to experience and disclose moral injury than male counterparts (Spilg et al. 2022; Zhizhong et al. 2020). Global mental health statistics also indicate that women are more likely to experience mental disorders than men (Shi et al. 2021). Women were almost three times as likely to experience common mental health issues than males (McManus et al. 2016).

The effects of moral injury among nurses include higher incidences of medical errors (Rodríguez et al. 2021). These errors have been reported to lead to insecurities, anxiety and a lack confidence in managing and resolving ethical dilemmas among student nurses (Hassanzadeh Naeini et al. 2020). Other implications include reduced quality of life, lower job satisfaction, absenteeism and decreased work productivity among nurses contributing to poor health system and patient outcomes (Rodríguez et al. 2021).

### Effects of moral injury on academic performance

The results further indicated that 44.0% of the respondents reported poor academic performance. The respondents in this study endorsed the items examining poor academic performance and admitted to facing academic problems. Most student nurses reported that the course was not what they had expected and found it too challenging. Half the student nurses found the course material uninteresting and the workload and pace too great. The causes that could have contributed to these problems were linked to pressures at home and work. This was evidenced by 62.1% of respondents reporting increased pressures and a temporary crisis at home and work.

Nursing course has a challenging, intensive and demanding curriculum that requires a lot of time and effort from the students (Manana et al. 2023). This affects their ability to balance their academic and social lives, and may limit their opportunities for leisure and relaxation. As a result, student nurses may experience high levels of burnout, fatigue, stress and anxiety, which can affect their well-being and academic performance.

The correlation between moral injury and academic performance was examined in this study. Even though academic performance had a positive weak linear relationship with moral injury (*r* = 0.196), there was a statistically significant relationship between the two after the four-symbol linear regression equation (*p* = 0.029). These results mean that moral injury has adverse effects on academic performance, suggesting that student nurses who suffer from moral injury face academic challenges.

Comparable statistics from the literature to gauge the magnitude of poor academic performance among student nurses affected by moral injury might be scarce. Nevertheless, Albright (2023) provided a qualitative comprehensive analysis of moral injury’s consequences on educators and their capacity to deliver high-quality academic material. When educators encounter moral injury, it can indirectly affect their students’ academic performance. This is an indirect relationship that moral injury has towards the individuals presenting with the symptoms.

The implications of poor academic performance in student nurses may increase the severity, frequency and rate of moral injury which can negatively affect the quality of care, motivation and career satisfaction of these students (Janatolmakan et al. 2021). Moral injury can also lead to an increase in dropout rates in undergraduate institutions and attrition rates of young nurses out of the profession (Kvitsiani et al. 2023). The nursing training programme has experienced a high attrition rate in recent years, with one-third of the students failing to complete because of the negative consequences of mental health (Manana et al. 2023). According to Jones-Berry (2021), some of the contributing factors to this phenomenon are the lack of prior experience, unfamiliarity with the clinical setting, insufficient knowledge base, difficulty in resolving ethical dilemmas and the discrepancy between theory and practice.

### Strengths and limitations

This study elucidated the prevalence of moral injury among student nurses at one nursing education institution. The effects of moral injury on nursing student nurses’ academic performance were also elucidated. The results could be used to guide the development of interventions to build moral resilience among student nurses. However, this study had some limitations that were evident from the data analysis of respondents’ demographic information, where correlation analysis was not performed between moral injury and the demographic variables of age, gender and level of study. Such an analysis would have revealed whether there were any differences or associations between these variables and moral injury, and thus inform the development of strategies for student nurses and professionals.

### Recommendations

Nursing education institutions should collaborate with Clinical Education and Teaching Units of the clinical facilities to ensure availability of platforms where students will be able to voice their concerns and encounters with immoral behaviours during their clinical placements. There is also a need for HEIs to design and implement a module that addresses the concepts of moral resilience, moral courage and ethics education, and regular assessments of moral and ethical competency in student nurses. These strategies will assist students in understanding the importance of compassion and responsibility towards vulnerable patients. When students are equipped with platforms where they will be able to voice their concerns about moral resilience, moral courage and moral and ethical competency, they will feel empowered to speak up against patient abuse and advocate for those patients who cannot defend themselves. This ability to voice concerns is essential for preventing harm for the patients and the student nurses through moral injury.

## Conclusion

This study aimed to investigate the prevalence and effects of moral injury among student nurses at a HEI in South Africa using MISS-HP questionnaire. The results indicated that a significant number of student nurses suffer from moral injury. The results further indicated that moral injury has a negative effect on student nurses’ academic performance.
